# Supplementary material for: Ablation of the Integrin CD11b Mac-1 Limits Deleterious Responses to Traumatic Spinal Cord Injury and Improves Functional Recovery in Mice
Source: Cells. 2024 Sep 20;13(18):1584. doi: 10.3390/cells13181584 (PMC11430243; doi:10.3390/cells13181584)
Supplement: Supplementary file 1 [file cells-13-01584-s001.zip › cells-3007002-supplementary.pdf]

**Ablation of the Integrin CD11b Mac-1 Limits Deleterious Responses to Traumatic Spinal Cord Injury and Improves Functional Recovery in Mice**

Yun Li [1], Zhuofan Lei [1], Rodney M. Ritzel [1,2], Junyun He [1], Simon Liu [1], Li Zhang [3], Junfang Wu [1,*]

[1]Department of Anesthesiology and Center for Shock, Trauma and Anesthesiology Research (STAR), University of Maryland School of Medicine, Baltimore, MD 21201, USA.

[2]Department of Neurology, McGovern Medical School, The University of Texas Health Science Center at Houston, Houston, TX 77030, USA.

[3]Department of Physiology, Center for Vascular and Inflammatory Diseases, University of Maryland School of Medicine, Baltimore, MD 21201, USA.

* Correspondence: junfang.wu@som.umaryland.edu Tel.: 1-410-706-5189

**Supplementary Information**

Supplemental Information includes Supplemental six figures and figure legends.

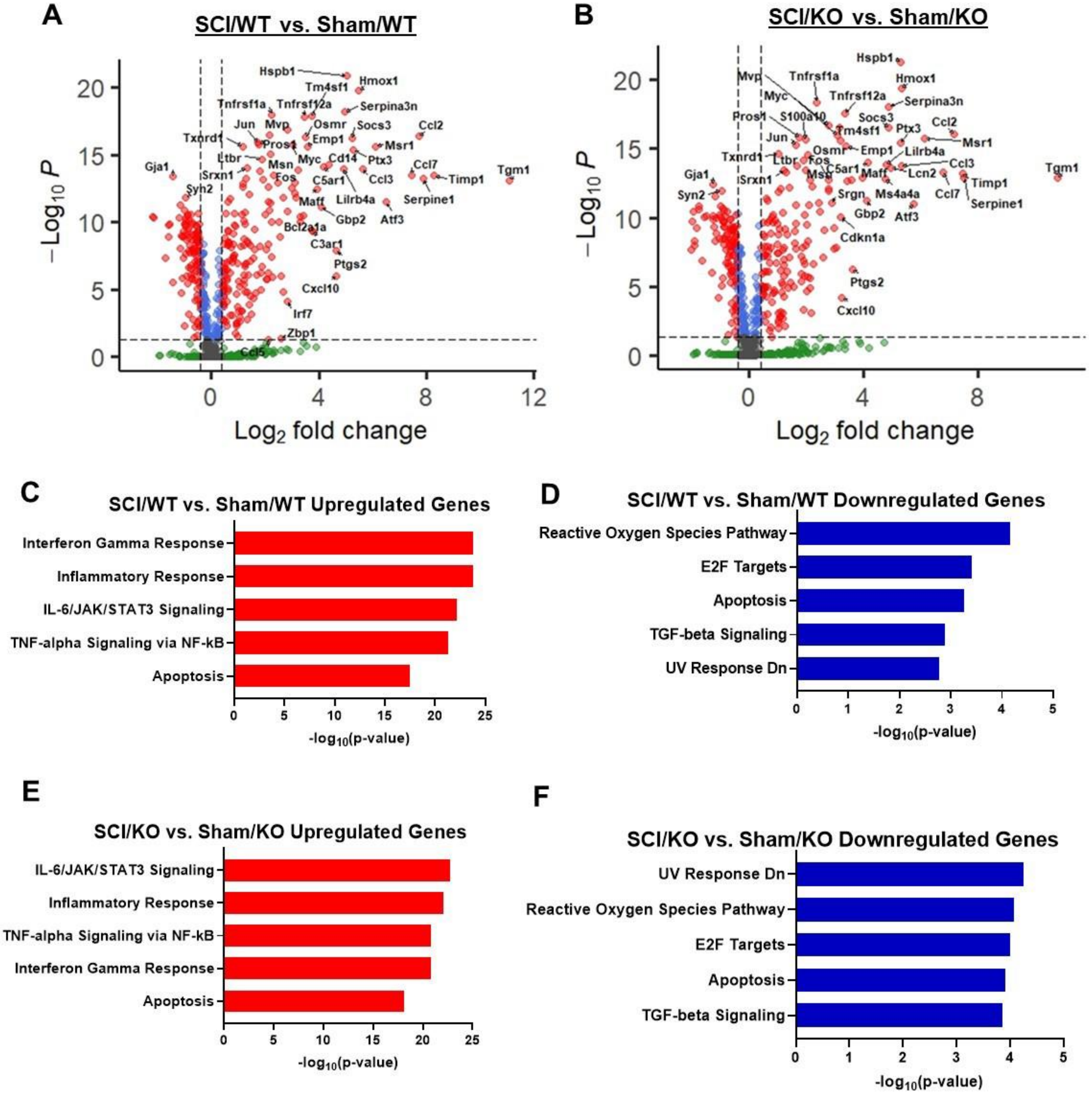


**Supplementary Figure S1. Genetic ablation of CD11b leads to activation of differential pathways at 1d after SCI. (A-B)** Volcano plot of SCI-induced DEGs in the spinal cord of C57BL/6 WT (A) and CD11b KO (B) mice at 1 d post-injury. **(C-F)** Pathway enrichment analysis of up and downregulated DEGs in WT and CD11b KO mice. n=6 mice/group.

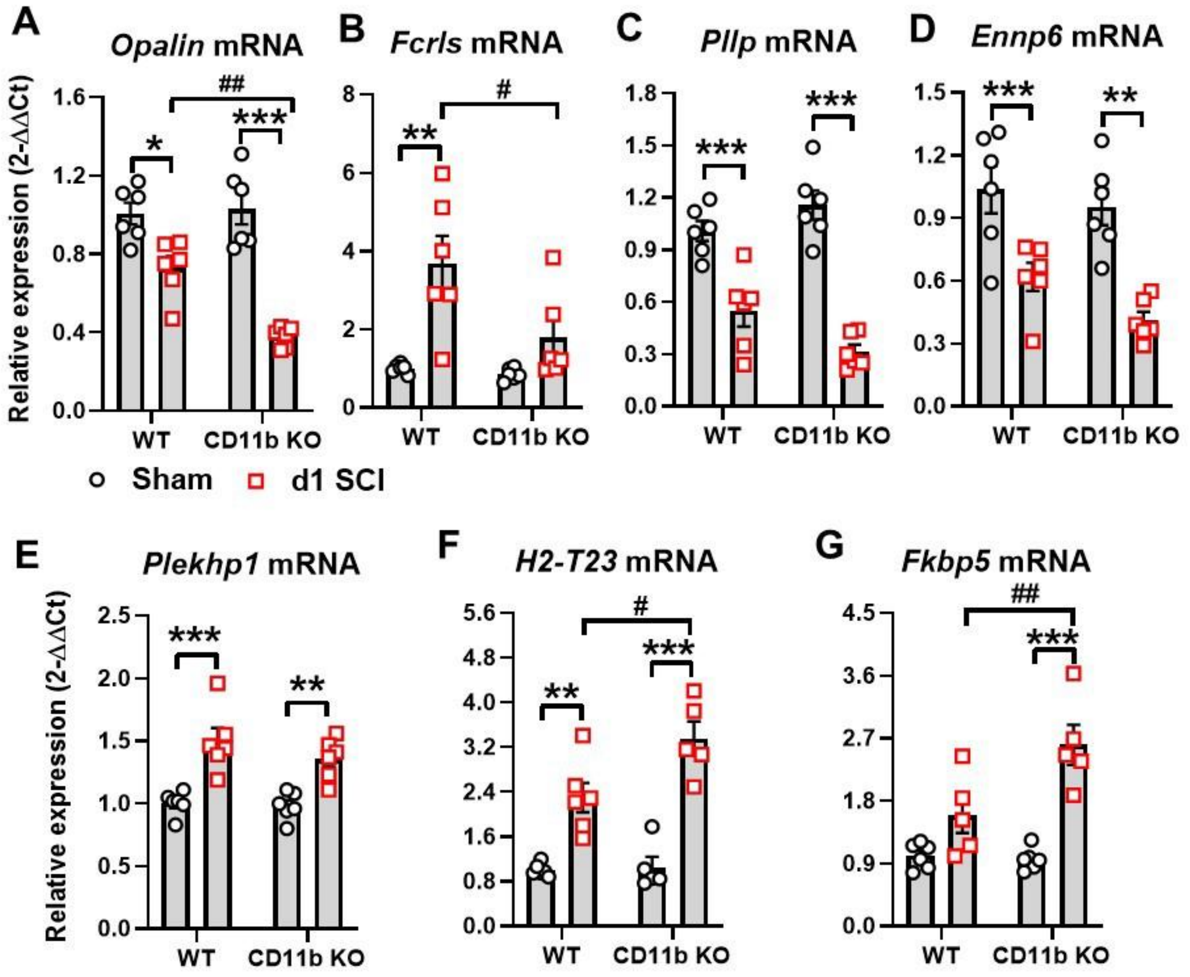


**Supplementary Figure S2. Ablation of CD11b leads to transcriptomic changes of inflammation-related genes at 1d after SCI.** qPCR analysis was used to examine the mRNA expression of *Opalin* **(A)**, *Fcrls* **(B)**, *Pllp* **(C)**, *Ennp6* **(D)**, *Plekhp1* **(E)**, *H2-T23* **(F)**, and *Fkbp5* **(G)**. *p<0.05, **p<0.01, ***p<0.001 vs. Sham groups. #p<0.05, ##p<0.01 vs. SCI/WT. n=5-6 mice/group. Two-way ANOVA followed by Tukey's post hoc test.

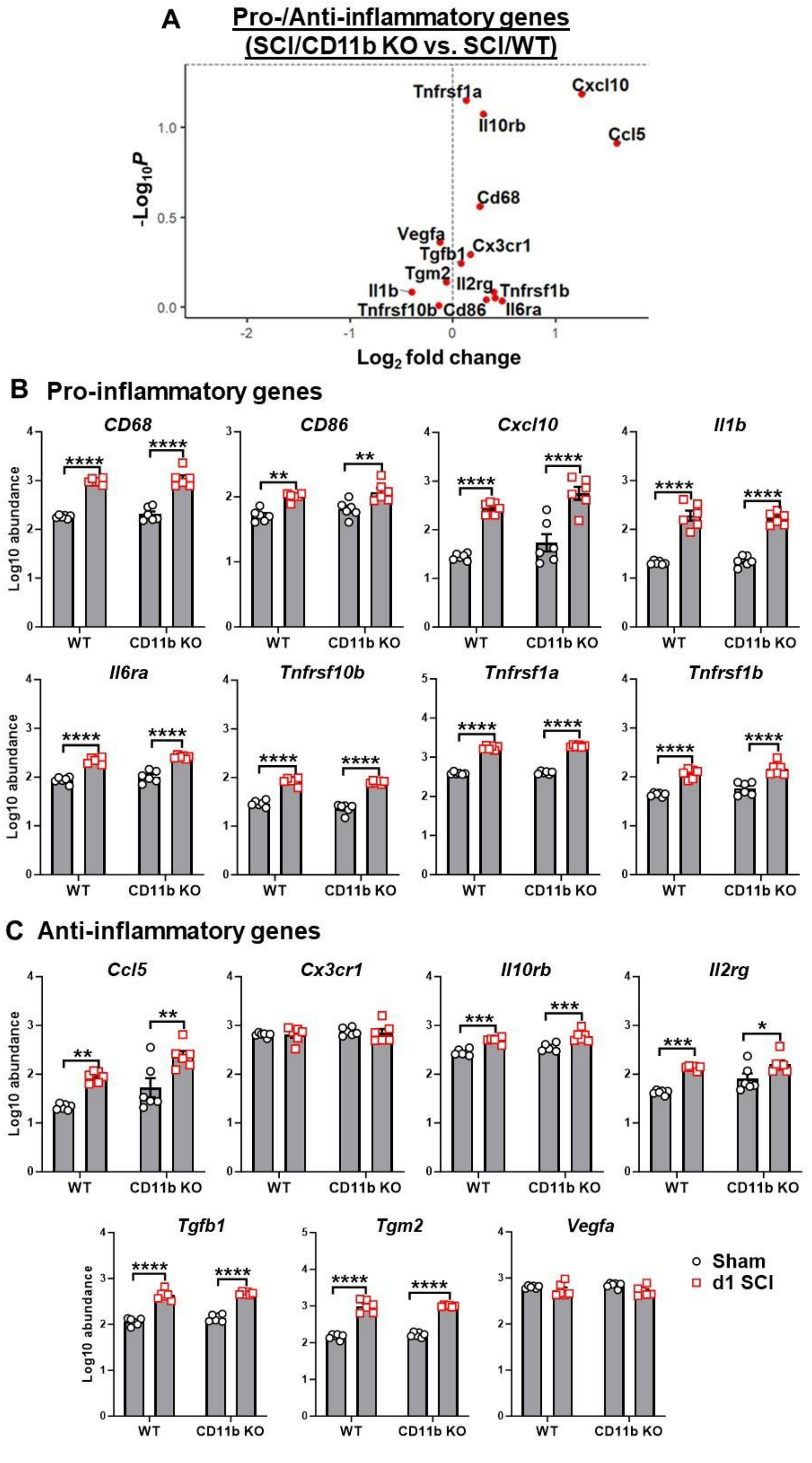


**Supplementary Figure S3. Ablation of CD11b has minimal effect on classic neuroinflammatory genes detected through NanoString**. The expression levels of pro- and anti-inflammatory genes were examined individually in NanoString and categorized into pro- and anti-inflammatory. (A) Volcano plot of selected pro-/anti-inflammatory after pairwise comparison of SCI/CD11b KO vs. SCI/WT. (B) Log10 abundance of pro-inflammatory genes. (C) Log10

abundance of anti-inflammatory genes. *p<0.05, **p<0.01, ***p<0.001, ****p<0.0001 vs. Sham groups. n=5-6 mice/group. Two-way ANOVA followed by Tukey's post hoc test.

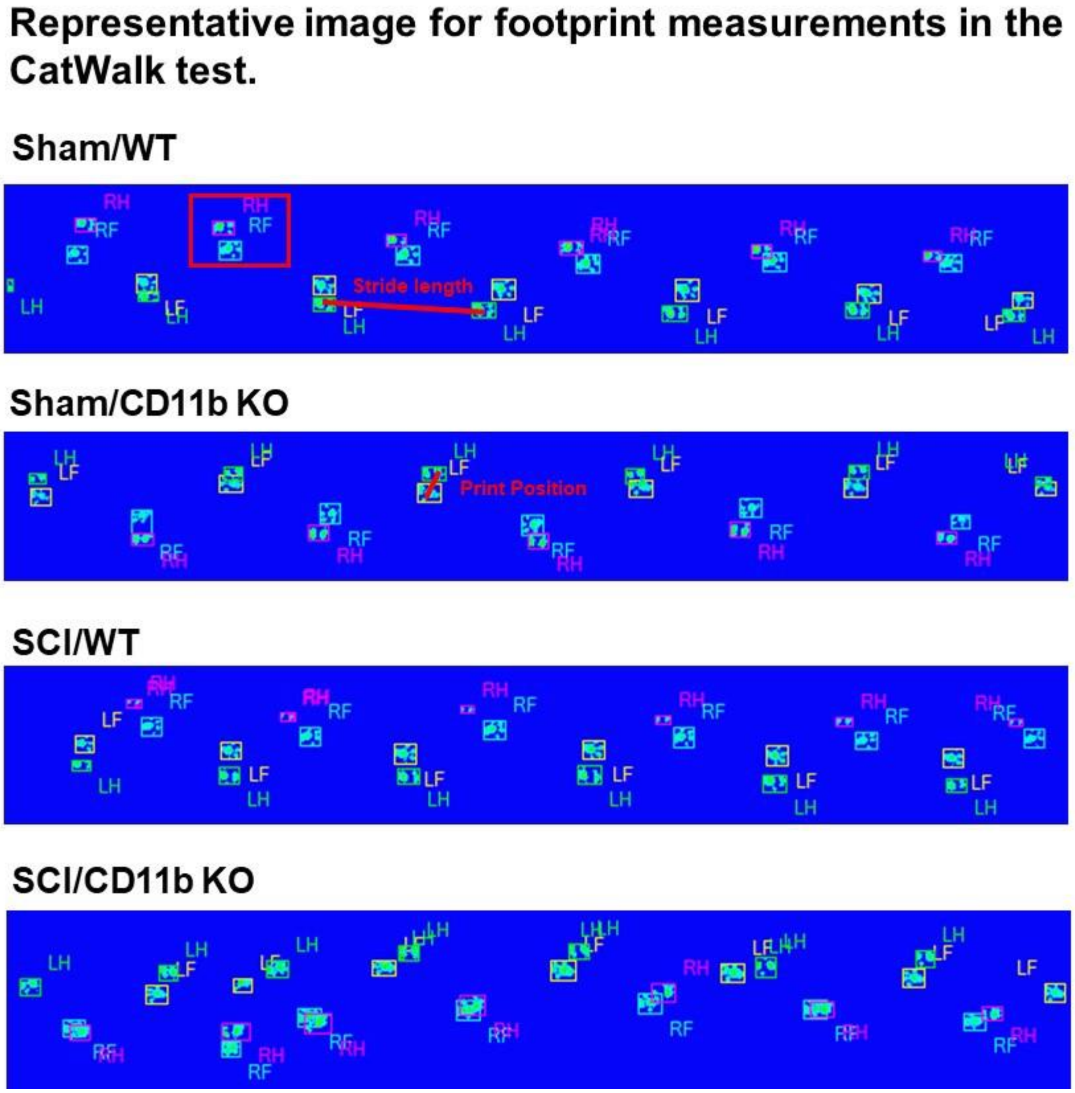


**Supplementary Figure S4. Representative images for footprint measurements in the CatWalk test.**

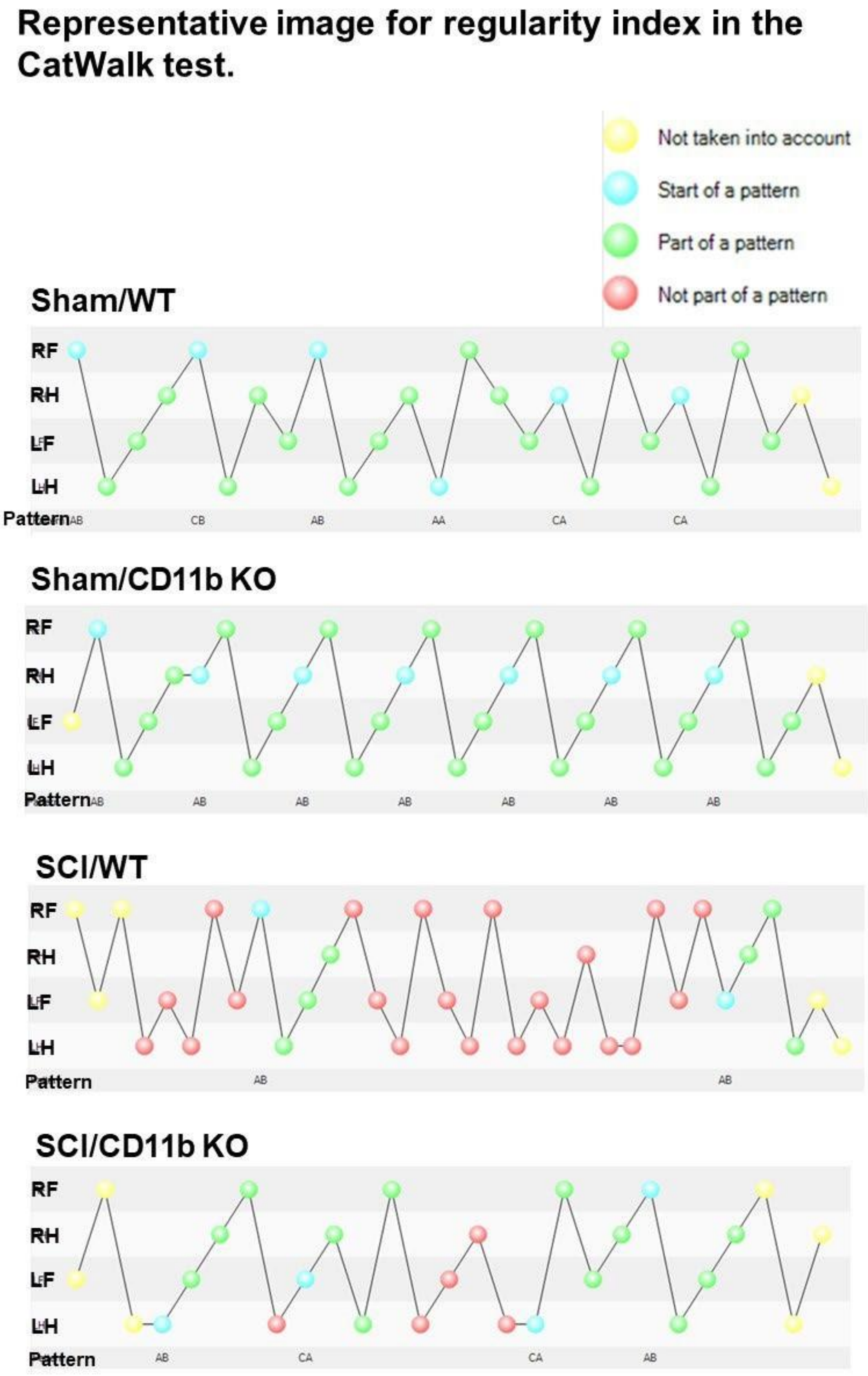


**Supplementary Figure S5. Representative images for regularity index in the CatWalk test.**

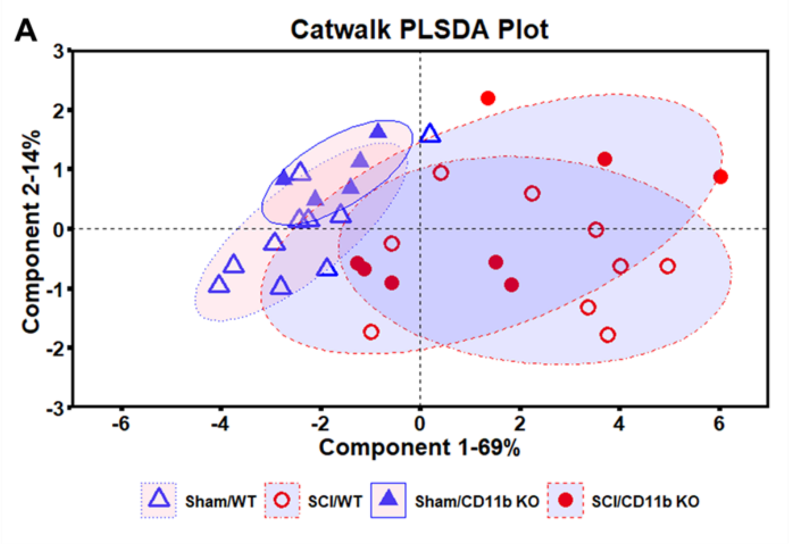


**B**

**Spearman Correlation Matrix**

| | Injury | Genotype | BMS | SL | PL | PW | SSRI | PD (RF->LH) | PD (LF->RH) | PP | BOS | PA | MCA | MCMI |
|---|---|---|---|---|---|---|---|---|---|---|---|---|---|---|
| Injury | 1.000 | 0.139 | -0.915 | -0.424 | -0.655 | -0.648 | -0.804 | 0.695 | 0.736 | 0.851 | -0.546 | -0.509 | -0.546 | -0.546 |
| Genotype | 0.139 | 1.000 | 0.033 | -0.162 | -0.148 | -0.065 | 0.038 | 0.045 | -0.003 | -0.038 | -0.231 | -0.169 | -0.159 | -0.224 |
| BMS | -0.915 | 0.033 | 1.000 | 0.453 | 0.764 | 0.755 | 0.877 | -0.735 | -0.765 | -0.872 | 0.686 | 0.645 | 0.679 | 0.665 |
| SL | -0.424 | -0.162 | 0.453 | 1.000 | 0.343 | 0.287 | 0.550 | -0.600 | -0.515 | -0.533 | 0.378 | 0.246 | 0.245 | 0.292 |
| PL | -0.655 | -0.148 | 0.764 | 0.343 | 1.000 | 0.911 | 0.707 | -0.478 | -0.638 | -0.693 | 0.619 | 0.954 | 0.960 | 0.942 |
| PW | -0.648 | -0.065 | 0.755 | 0.287 | 0.911 | 1.000 | 0.737 | -0.538 | -0.552 | -0.745 | 0.533 | 0.922 | 0.934 | 0.875 |
| SSRI | -0.804 | 0.038 | 0.877 | 0.550 | 0.707 | 0.737 | 1.000 | -0.758 | -0.699 | -0.887 | 0.681 | 0.594 | 0.609 | 0.614 |
| PD (RF->LH) | 0.695 | 0.045 | -0.735 | -0.600 | -0.478 | -0.538 | -0.758 | 1.000 | 0.567 | 0.716 | -0.445 | -0.405 | -0.421 | -0.446 |
| PD (LF->RH) | 0.736 | -0.003 | -0.765 | -0.515 | -0.638 | -0.552 | -0.699 | 0.567 | 1.000 | 0.672 | -0.675 | -0.487 | -0.510 | -0.548 |
| PP | 0.851 | -0.038 | -0.872 | -0.533 | -0.693 | -0.745 | -0.887 | 0.716 | 0.672 | 1.000 | -0.571 | -0.572 | -0.597 | -0.562 |
| BOS | -0.546 | -0.231 | 0.686 | 0.378 | 0.619 | 0.533 | 0.681 | -0.445 | -0.675 | -0.571 | 1.000 | 0.550 | 0.551 | 0.541 |
| PA | -0.509 | -0.169 | 0.645 | 0.246 | 0.954 | 0.922 | 0.594 | -0.405 | -0.487 | -0.572 | 0.550 | 1.000 | 0.996 | 0.948 |
| MCA | -0.546 | -0.159 | 0.679 | 0.245 | 0.960 | 0.934 | 0.609 | -0.421 | -0.510 | -0.597 | 0.551 | 0.996 | 1.000 | 0.951 |
| MCMI | -0.546 | -0.224 | 0.665 | 0.292 | 0.942 | 0.875 | 0.614 | -0.446 | -0.548 | -0.562 | 0.541 | 0.948 | 0.951 | 1.000 |

-1.0 -0.5 0 0.5 1.0

**Supplemental Figure S6. Correlation analysis of Genotype and Injury with Catwalk gait/print parameters. (A)** PLSDA was performed with all stance and footprint parameters from Catwalk gait analysis. The two main components of variation were captured on the x- and y-axis and showing a clear separation of clusters between the injury and genotype groups. **(B)** Matrix of correlation coefficients obtained from pairwise Spearman analysis. Catwalk parameters include Stride Length (SL), Print Length (PL), Print Width (PW), Step Sequence Regularity Index (SSRI), Phase dispersions (PD), Base of Support (BOS), PA (Print Area), MCA (Max Contact Area) and MCMI (Max Contact Max Intensity). All Catwalk parameters were included with the added rank variable of Injury (0=Sham, 1=SCI), Genotype (0=WT, 1=CD11b KO), and the final BMS scores at 6w post-injury.
